# Supplementary material for: Synergistic Photocatalytic Oxidation and Reductive Activation of Peroxymonosulfate by Bi-Based Heterojunction for Highly Efficient Organic Pollutant Degradation
Source: Nanomaterials (Basel). 2025 Mar 20;15(6):471. doi: 10.3390/nano15060471 (PMC11944557; doi:10.3390/nano15060471)
Supplement: Supplementary file 1 [file nanomaterials-15-00471-s001.zip › nanomaterials-3535688-supplementary.pdf]

Synergistic Photocatalytic Oxidation and Reductive Activation of Peroxymonosulfate by Bi-based Heterojunction for Highly Efficient Organic Pollutant Degradation

Xiao-Peng Zhao[#1], Yang Wang[#1,2], Fang-Ning Liu[1], Xiao-Bin Ye[1], Shang-Xiong Wei[1], Yi-Lin Sun[1], Jing-Hui He[1,3*]

[#] These authors contributed equally to this work

[1] College of Chemistry, Chemical Engineering and Materials Science, Soochow University, Suzhou, Jiangsu 215123, China.

[2] State key Laboratory of silicate materials for architectures, Wuhan University of Technology, Wuhan, Hubei 430070, China.

[3] Advanced Water Technology Laboratory, National University of Singapore (Suzhou) Research Institute, Suzhou, Jiangsu 215123, China

1. Chemicals

Sodium acetate, $Bi(NO_3)_3 \cdot 5H_2O$, NH_4VO_3, $Cu(NO_3)_2 \cdot 3H_2O$, NaOH, nitric acid, and ammonia were purchased from Shanghai Aladdin Biochemical Technology Co., Ltd. Anhydrous acetonitrile, ethanol, o-dichlorobenzene, n-butanol, tetrahydrofuran, and glacial acetic acid were obtained from Sinopharm Group. Methyl blue, tetracycline, oxytetracycline, norfloxacin, levofloxacin, and rhodamine B were purchased from Shanghai Macklin Biochemical Technology Co., Ltd. All reagents were used directly without further purification.

2. Instruments

X-ray diffraction (XRD) patterns were carried out on a Bruker D8 Advance Diffractometer with a Cu Kα source (λ = 0.1541 nm). X-ray photoelectron spectroscopy (XPS) was conducted using monochromatic Al Kα radiation on an ESCALAB 250Xi. In situ irradiation XPS measurements were performed under similar conditions with the introduction of ultraviolet–visible light (λ = 365 nm, 300 W). Transmission electron microscopy (TEM) images were collected by TecnaiG2F20. Scanning electron microscopy (SEM) images of the materials were collected using Ragulus 8230. High-angle annular dark-field image (HAADF) mapping images were collected using the Talos F200X system. Fourier transform infrared spectrometer was used to determine the specific functional groups of the catalyst. Ultraviolet–visible spectroscopy was measured by Shimadzu UV3600. Photoluminescence (PL) spectra were tested using Edinburgh FLS980. Edinburgh's FS5 was used to detect fluorescence time-resolved photoluminescence (TRPL) spectra. Photoelectrochemical tests were carried out through a CorrTest CS310 electrochemical workstation. Electrochemical impedance spectroscopy (EIS) analysis of the catalyst was conducted using a CorrTest CS310 electrochemical workstation. The alternating current was set to 10 mV, and the frequency range was from 100,000 to 1 Hz. Electron paramagnetic resonance (EPR) signals were measured by an EPRspectrometer (JEOL JES-X320). Intermediate products were analyzed by liquid chromatography–mass spectrometry (LC-MS) (Bruker, MicroQ-TOF).

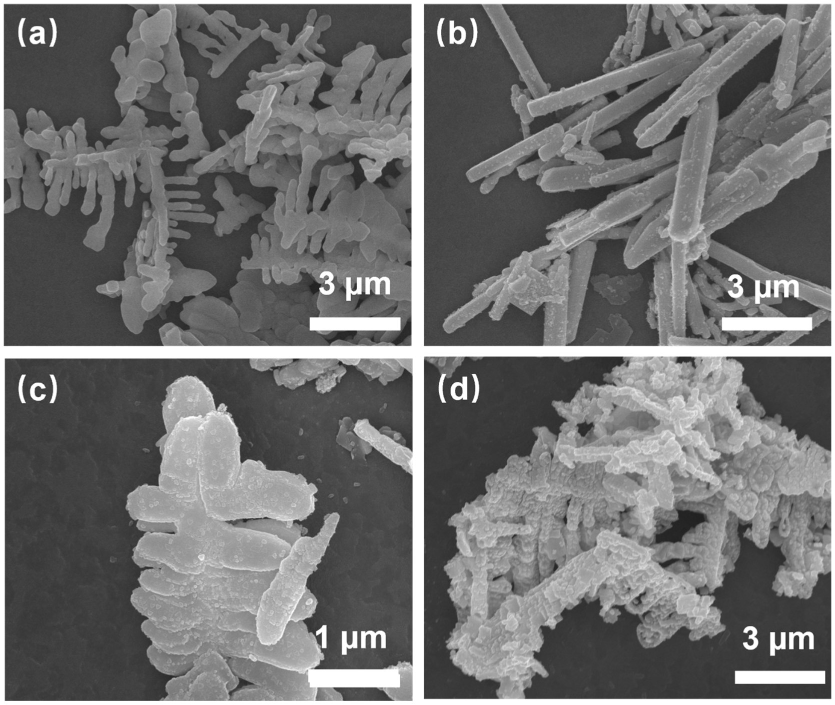

Figure S1. The SEM images of (**a**) $BiVO_4$, (**b**) $CuBi_2O_4$, (**c**) 0.2-$CuBi_2O_4$/ $BiVO_4$, and (**d**) 0.5-$CuBi_2O_4$/ $BiVO_4$.

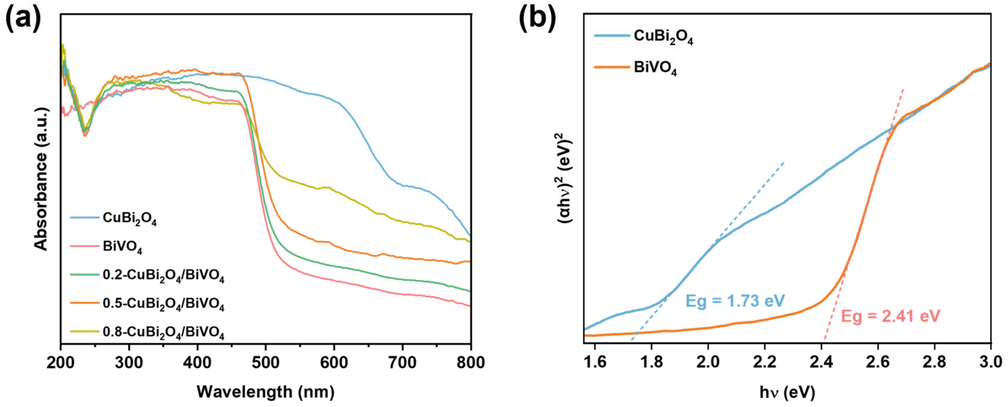

Figure S2. (**a**) The ultraviolet–visible diffuse reflectance spectrogram of the catalyst. (**b**) The Tacu plots of $CuBi_2O_4$ and $BiVO_4$.

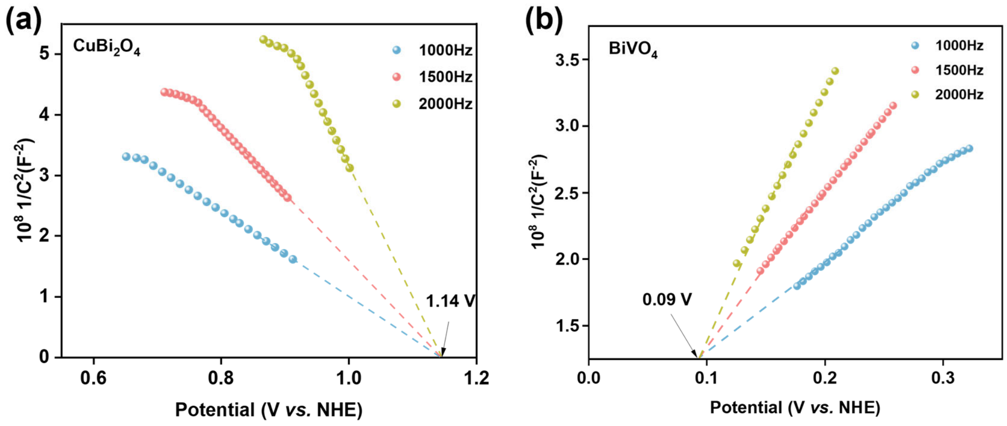

Figure S3. (**a**) The Mott–Schottky curve of $CuBi_2O_4$. (**b**) The Mott–Schottky curve of $BiVO_4$.

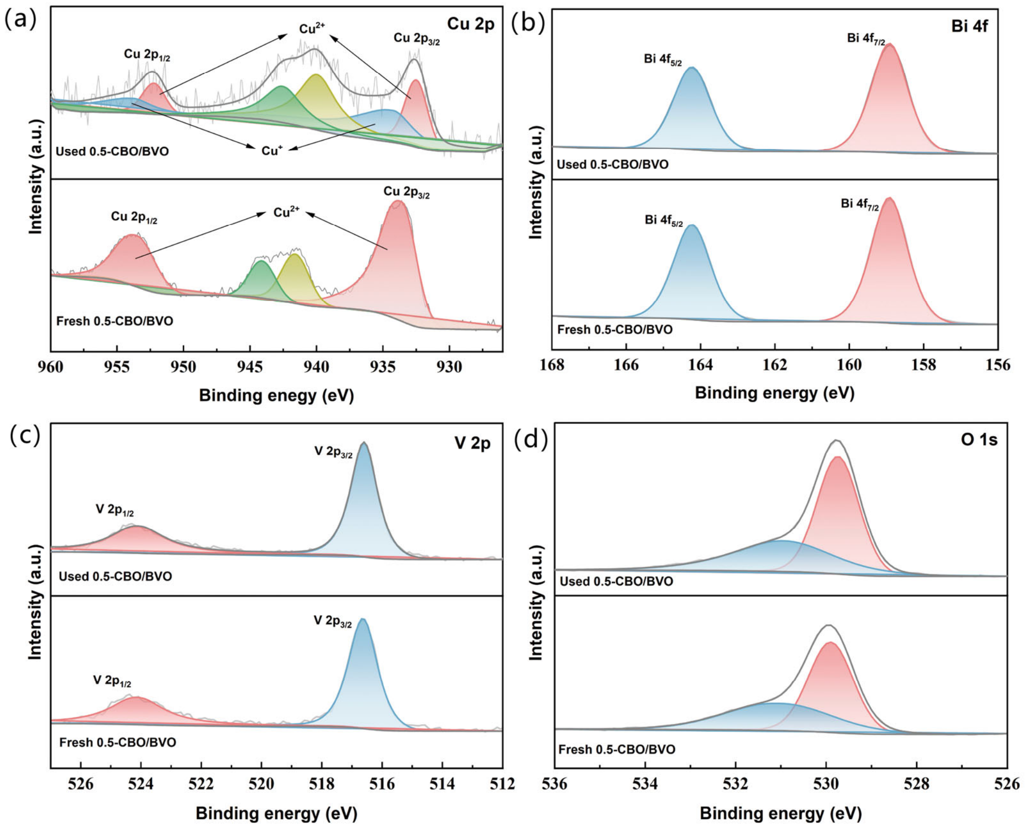

Figure S4. The XPS spectra of (**a**) Cu 2p, Bi 4f, (**b**) V 2p, and (**c**) O 1s before and (**d**) after the 0.5-$CuBi_2O_4/BiVO_4$ reaction.

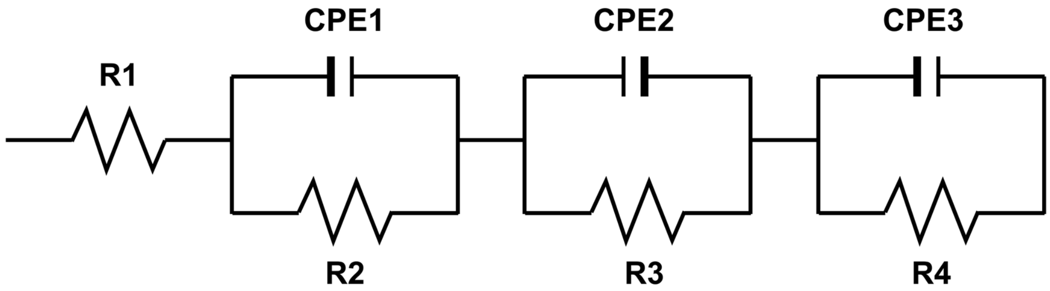

Figure S5. EIS-analyses of 0.5-$CuBi_2O_4$/$BiVO_4$ using equivalent circuits.

Parameter	Error
Resistance 1	7.752
Capacitance 1	13..78
Resistance 1	6.232
Capacitance 2	1.692
Resistance 2	0.6952
Capacitance 3	8.361
Resistance 3	14.02

Table S1. The fitted parameters of the equivalent circuits.

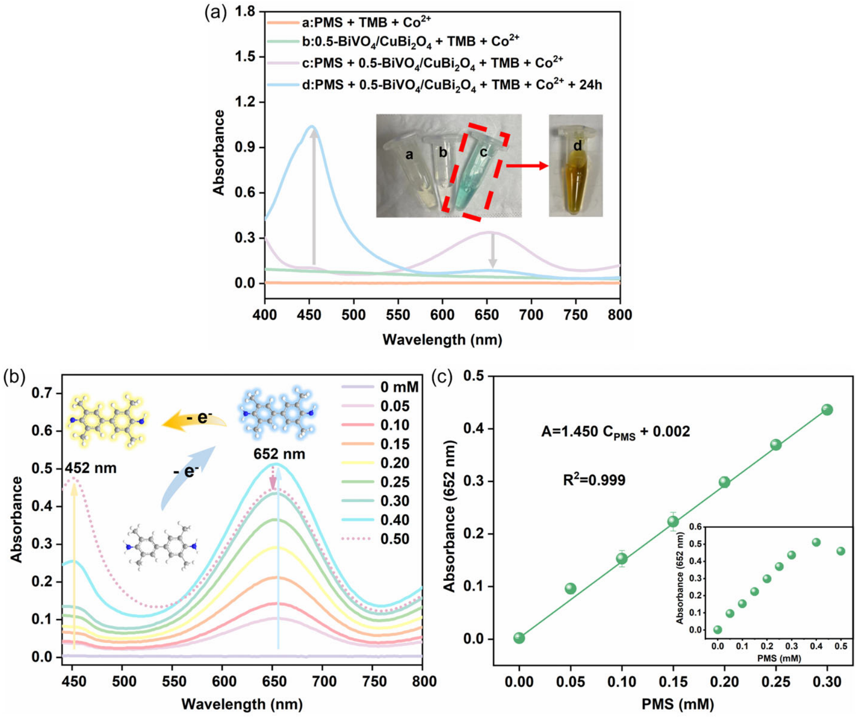

Figure S6. (**a**) Absorption curves of different solutions, (**b**) absorption curves of solutions in the presence of PMS at different concentrations (ranging from 0 to 0.5 mM), and (**c**) absorbance at 652 nm (the inset shows the photos of the corresponding reaction solutions).

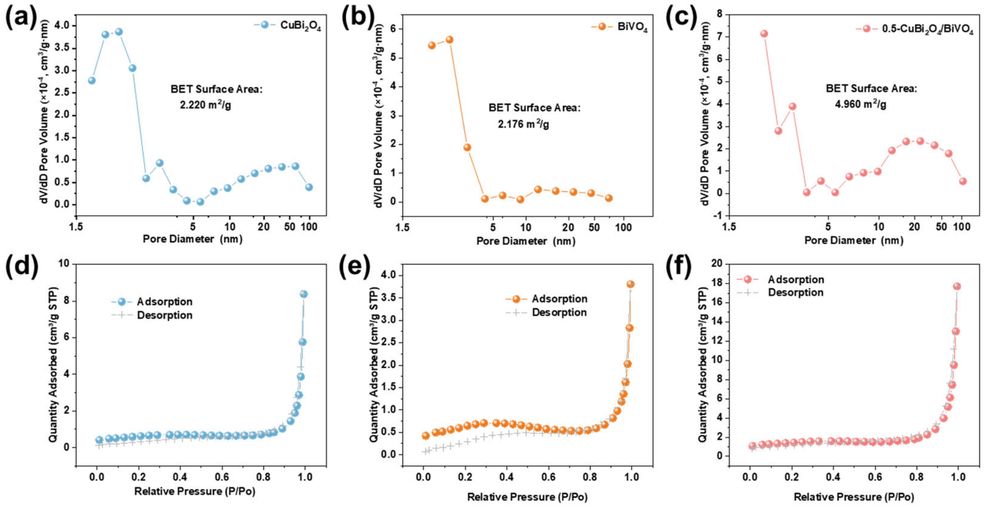

Figure S7. (**a**) Pore size distribution diagram and (**d**) N_2 adsorption–desorption curve of $CuBi_2O_4$. (**b**) Pore size distribution diagram and (**e**) N_2 adsorption–desorption curve of $BiVO_4$. (**c**) Pore size distribution diagram and (**f**) N_2 adsorption–desorption curve of 0.5-$CuBi_2O_4$/$BiVO_4$. (Samples have small surface areas (<10 m^2/g); thus, possible crossing due to measuring error is plausible. Given the absence of additional energy barriers during gas adsorption and desorption processes, no significant hysteresis phenomenon is observed in the isotherms [1-3]. This does not affect the experimental results.)

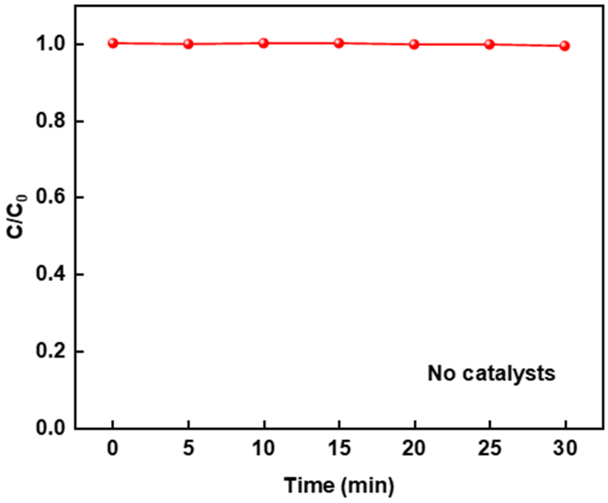

Figure S8. Removal efficiency of ciprofloxacin in the presence of only visible light and PMS.

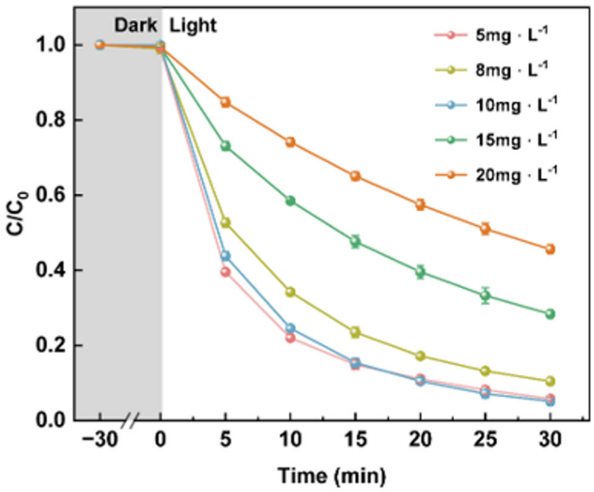

Figure S9. The effects of different initial ciprofloxacin concentrations.

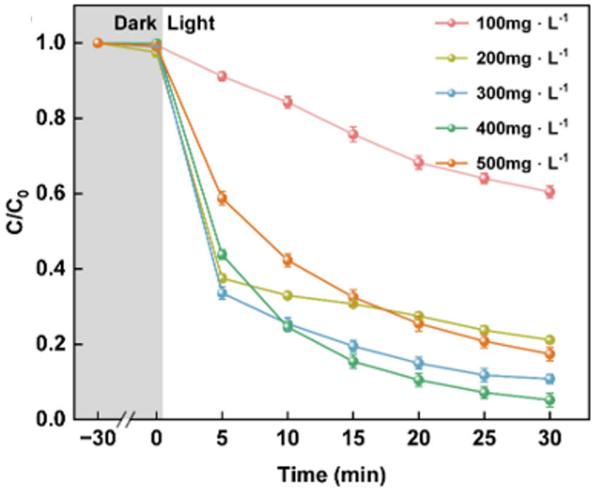

Figure S10. The effects of different dosages of catalyst used.

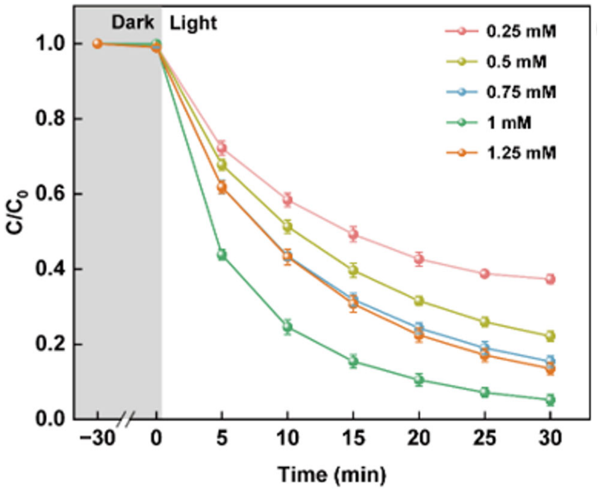

Figure S11. The effects of different PMS concentration.

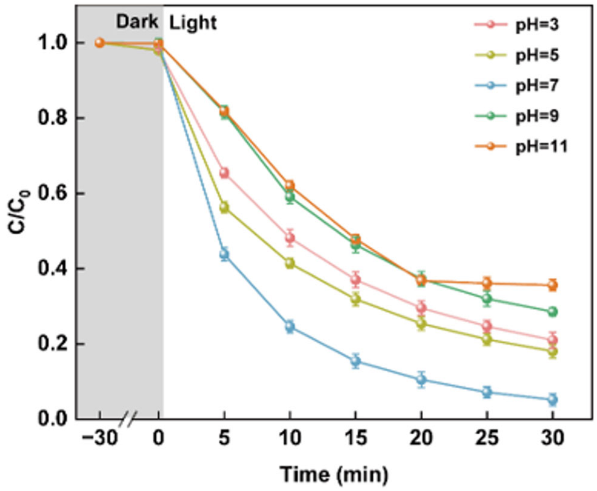

Figure S12. The effects of different pH values.

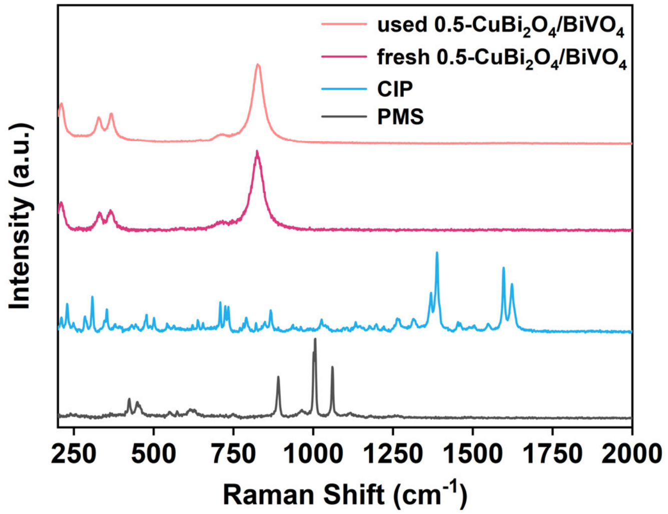

Figure S13. The Raman spectra of fresh 0.5-$CuBi_2O_4/BiVO_4$, used 0.5-$CuBi_2O_4/BiVO_4$ without wash, CIP, and PMS.

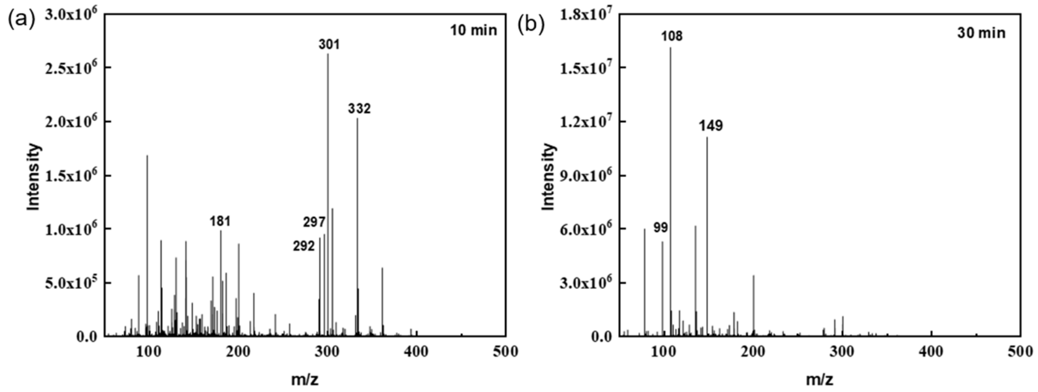

Figure S14. LC-MS plots of degraded ciprofloxacin at (**a**) 10 min and (**b**) 30 min.

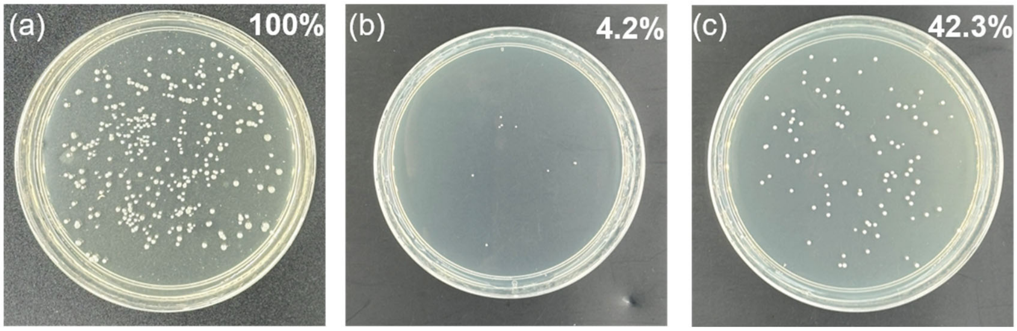

Figure S15. (**a**) Blank control. Images of *E. coli* growth (**b**) before and (**c**) after ciprofloxacin degradation.

References

1. H. Cui, N. Xing, A green method for the preparation of nitrogen-doped mesoporous carbon and its application in adsorptive desulfurization. *New J. Chem.* **2024**, *48*, 15802-15809
2. J. Wang, Q. Liu, An efficient one-step condensation and activation strategy to synthesize porous carbons with optimal micropore sizes for highly selective CO_2 adsorption†. *Nanoscale* **2014,** *6*, 4148-4156.
3. A.B. Fuertes, P. Valle-Vigón, M. Sevilla, Synthesis of colloidal silica nanoparticles of a tunable mesopore size and their application to the adsorption of biomolecules. *J. Colloid and Interface Sci.* **2010,** *349*, 173-180.
